# "Direct DICOM Slice Landmarking” A Novel Research Technique to Quantify Skeletal Changes in Orthognathic Surgery

**DOI:** 10.1371/journal.pone.0131540

**Published:** 2015-08-07

**Authors:** Anas Almukhtar, Balvinder Khambay, Ashraf Ayoub, Xiangyang Ju, Ali Al-Hiyali, James Macdonald, Norhayati Jabar, Tazuko Goto

**Affiliations:** 1 The Dental School, MVLS College, University of Glasgow, Glasgow, United Kingdom; 2 Paediatric Dentistry and Orthodontics, Faculty of Dentistry, The University of Hong Kong, Hong Kong, P R China; 3 Medical Device Unit, Department of Clinical Physics and Bioengineering, NHS Greater Glasgow and Clyde, Glasgow, United Kingdom; University of North Carolina at Chapel Hill, UNITED STATES

## Abstract

The limitations of the current methods of quantifying the surgical movements of facial bones inspired this study. The aim of this study was the assessment of the accuracy and reproducibility of directly landmarking of 3D DICOM images (Digital Imaging and Communications in Medicine) to quantify the changes in the jaw bones following surgery. The study was carried out on plastic skull to simulate the surgical movements of the jaw bones. Cone beam CT scans were taken at 3mm, 6mm, and 9mm maxillary advancement; together with a 2mm, 4mm, 6mm and 8mm “down graft” which in total generated 12 different positions of the maxilla for the analysis. The movements of the maxilla were calculated using two methods, the standard approach where distances between surface landmarks on the jaw bones were measured and the novel approach where measurements were taken directly from the internal structures of the corresponding 3D DICOME slices. A one sample t-test showed that there was no statistically significant difference between the two methods of measurements for the y and z directions, however, the x direction showed a significant difference. The mean difference between the two absolute measurements were 0.34±0.20mm, 0.22±0.16mm, 0.18±0.13mm in the y, z and x directions respectively. In conclusion, the direct landmarking of 3D DICOM image slices is a reliable, reproducible and informative method for assessment of the 3D skeletal changes. The method has a clear clinical application which includes the analysis of the jaw movements “orthognathic surgery” for the correction of facial deformities.

## Introduction

Previous studies have reported on the three-dimensional changes of the skeletal hard tissue following orthognathic surgery. The outcome can be categorised into four main groups; those using cephalometeric analysis, principal component analysis (PCA), volumetric changes and colour error maps.

Lateral cephalometric analysis assessed changes in linear and angular measurements. Despite the shortcomings of capturing 2D data and landmark identification [1], it is still routinely used for the evaluation of sagittal changes in maxillary and mandibular position [2–4]. Three-dimensional (3D) cephalometry on the other hand potentially facilitates a more comprehensive analysis of the craniofacial morphology in which the anatomical landmarks are placed on the skeletal 3D surface model constructed from a CT scan [5],. However, many of these landmarks are placed on anatomical features that are difficult to reproduce or may have changed during surgery. In addition the accuracy of the 3D surface model is questionable due to the conversion from volumetric data to surface model being dependent on the user-selected Hounsfield unit (HU) value[6].

A recent study demonstrated the use of complex morphometric analysis involving generalized Procrustes analysis (GPA), grids of thin-splines and principle component analysis (PCA) to allow visualisation of hard tissue change [7]. The outcome of this form of analysis can be used to explain variation of the data based on PCA rather than actual 3D movements. The translation and interpretation of this information into a clinical setting seems to be too complicated for routine use.

Volumetric changes have been used to analyse hard tissue displacement after orthognathic surgery [8]. Following superimposition of the pre- and post-operative images, and subtraction of the two images it is possible to calculate the volume differences in the corresponding structures. Although this method quantifies the changes in volume of the hard tissues following surgery, post-operative positional changes cannot be quantified by this method.

Colour coded distance maps or error maps are analytical tool incorporated in most CAD/CAM software packages to measure the relative distance between two 3D surface meshes. This is achieved by measuring the shortest distance from one vertex of one mesh to a second vertex on the second mesh. The measurements are visualised by changing the colour of the vertices of the meshes according to a colour scale, for example blue represents a negative measurement or reduction in size to red colour representing positive measurements or an increase in size [9–11].

This method uses the whole hard tissue surface area for calculating the magnitude of the movement and provides a single numerical value that can be used for comparisons. The major shortcoming of this method is that it measures the shortest distance between vertices of the adjacent meshes. These may not necessarily represent corresponding anatomical points. Depending on the clinical cases, this may affect the validity of the measurements. In fact a recent study has shown underestimation of the magnitude of change of simulated surgical movement by about 50–70% [12].

The limitations of the current methods of quantifying the hard tissue changes following the surgical correction of dentofacial deformities inspired the development of a novel method of measurement based on direct landmarking of the 3D DICOM images.

### Aims

To assess the accuracy and reproducibility of directly landmarking DICOM image slices and to quantify the positional change of the maxilla and the mandible following simulated orthognathic surgery.

## Materials and Methods

### Ethics

The study was conducted on a plastic skull model so no ethics approval was required, for the clinical validation West of Scotland Research Ethics approval was obtained (12/WS/0133) and the analysed patient records were anonymized

### Measurements of simulated surgical movement

Based on a previously reported technique a plastic skull was used to simulate the surgical movements and produce a reference measurement set [12]. The technique allowed movement of the maxilla and mandible in the anterior-posterior and vertical direction only with minimal lateral or rotation movement. Following a Le Fort I osteotomy and detachment of the maxilla from the skull three 5mm diameter spherical plaster markers were secured, using sticky wax, at the right greater palatine (GPR), left greater palatine (GPL) and the incisive foramina (IF). The maxilla was repositioned in the pre-osteotomy orientation using a template and secured to the skull using sticky wax. The template was removed once the maxilla was firmly attached to the skull.

To assess mandibular changes, a bilateral sagittal split osteotomy (BSSO) was performed on the plastic mandible and reassembled using two 3mm stainless steel screws per side. Five plaster sphere markers were placed on the mandible using sticky wax; left and right lingual foramen (LL and RL), left and right mental foramen (LM and RM) and genial tubercle (Ling).

The skull and maxilla were secured to a universally adjustable camera mount fixed to a 20mm thick acrylic base. A standard Denar slidematic facebow (Whipmix, Louisville, KY) was fitted and secured with laboratory putty were necessary and a circular spirit level placed on the anterior region of the face bow. By adjusting the camera’s universal joint, using the face bow and spirit level assembly, it was possible to orientate the Frankfort plane of the skull to true horizontal.

An adjustable stage was secured to the acrylic base below the level of the maxilla. The adjustable stage allowed movement in the sagittal direction only, whilst the adjustable platform controlled vertical movement by removing or adding “spacers” of known thickness.

The skull and facebow were secured to the acrylic base was positioned in the cone beam CT (CBCT) scanner (iCAT, Imaging Science, Hatfield). The skull’s position was re-checked and immediately prior to the CBCT scan the face bow was removed. A 22cm Extended Field Of View (EFOV) scan at 0.4mm voxel resolution was carried out. This baseline scan correctly orientated skull data was then saved as a DICOM file.

### Simulated maxillary and mandibular movements

The maxilla was secured to the adjustable platform using sticky wax; and released from the base of the skull. Using the adjustable stage and platform the maxilla was moved to the desired sagittal and vertical position. Sagittal measurements were taken using a Vernier caliper mounted perpendicular to the acrylic base. Vertical measurements were recorded using a vertical height caliper (Chesterman, Sheffield, UK) with an analogue Vernier scale with a known accuracy of 0.5 mm. The maxilla was then re-attached to the skull and then released from the adjustable platform. Cone beam CT scans using the EFOV option and 0.4mm voxel resolution were taken at 3mm 6mm and 9mm maxillary advancement; together with a 2mm, 4mm, 6mm and 8mm “downgraft” for each 3mm increment of maxillary advancement. In total, 12 different positions of the maxilla were recorded and each scan saved as a DICOM file.

The mandible was re-attached to the skull with the lower border of the mandible secured to the adjustable platform. Following removal of the screws the anterior segment of the mandible was translated forward and re-attached with sticky wax to the maxilla. Cone beam CT scans following the previous protocol were taken at 4mm, 6mm, 8mm and 10mm mandibular advancements.

To accurately determine maxillary and mandibular movement each DICOM image was converted to a surface mesh using MeVisLab (MeVis Medical Solutions Ltd., Germany) and saved in STL format. The original correctly oriented skull STL file was loaded into VRMesh (Seattle City, U.S.A) and all the remaining STL files were aligned to this baseline skull using the anterior cranial base as a common area of superimposition. Each image was re-saved in its new aligned 3D position. Using Minimagics (Materialise, Belgium) it was possible to import and align two STL files, create a profile of both images and then measure the sagittal and vertical distances between the two profiles at any point. This was performed for all the maxillary and mandibular movements. In total 4 down graft, 11 advancement and 12 combined down graft and advancement measurement were made for the maxilla and 4 advancement measurements for the mandible.

### 3D measurements directly landmarking the DICOM slices

The measurements were made using OnDemand3D software (Cybermed, Seoul, South Korea). The landmarking procedure involved three steps: pre-and post-operative DICOM image superimposition; 3D image orientation and creation of reference planes (x, y and z planes) and lastly a modified 3D cephalometeric analysis of the orthogonal measurements of 8 landmarks placed on the DICOM image slices. These steps were followed by calculation of the actual three-dimensional movement of each landmark due to changes in jaw position.

### DICOM image superimposition

**Fig 1:**
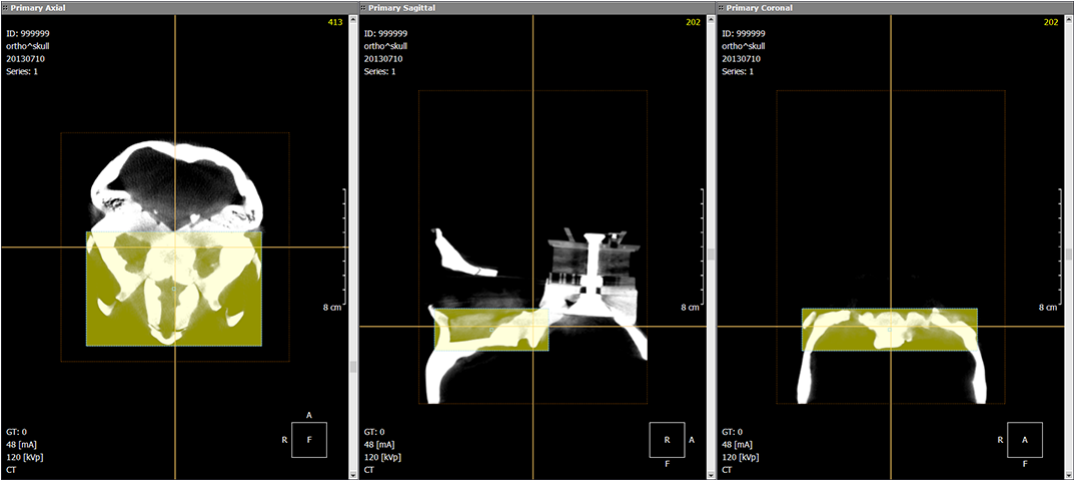
Voxel based registration, volume of interest selected on the anterior cranial base of the plastic skull.

The pre- and post-operative CBCT scanned DICOM images were imported into OnDemand3D software. Superimposition of the two images was accomplished using voxel-based registration. This involved two steps: manual alignment of the two images followed by automatic registration. The registration process was a series of iterative movement aimed at achieving the “best fit” based on the grey scale intensity between the two overlapping images, voxel by voxel(13). The region of interest for superimposition was the anterior cranial base as this was a stable and unaffected by surgery based on previously published studies [10–13], Fig 1. The grouped pre- and post-operative registered DICOM images were then re-oriented to the same horizontal position as the original plastic skull using right and left (porion) and the right (orbitalae) landmarks.

### Creation of reference planes

Following superimposition of the pre-operative and post-operative DICOM images a common reference plane could be constructed. Three reference planes were created; a horizontal (axial) plane formed by the two porion and the right orbital landmarks to mimic the surgical simulation setup, a sagittal (medial) plane was created by the nasion and sella landmarks and oriented perpendicular to the first plane, and a vertical (coronal) plane created perpendicular to both planes passing through sella. The x, y and z co-ordinates of any landmark placed on any of the DICOM slices could then be extracted orthogonally from these three planes.

### Orthogonal measurements

**Fig 2:**
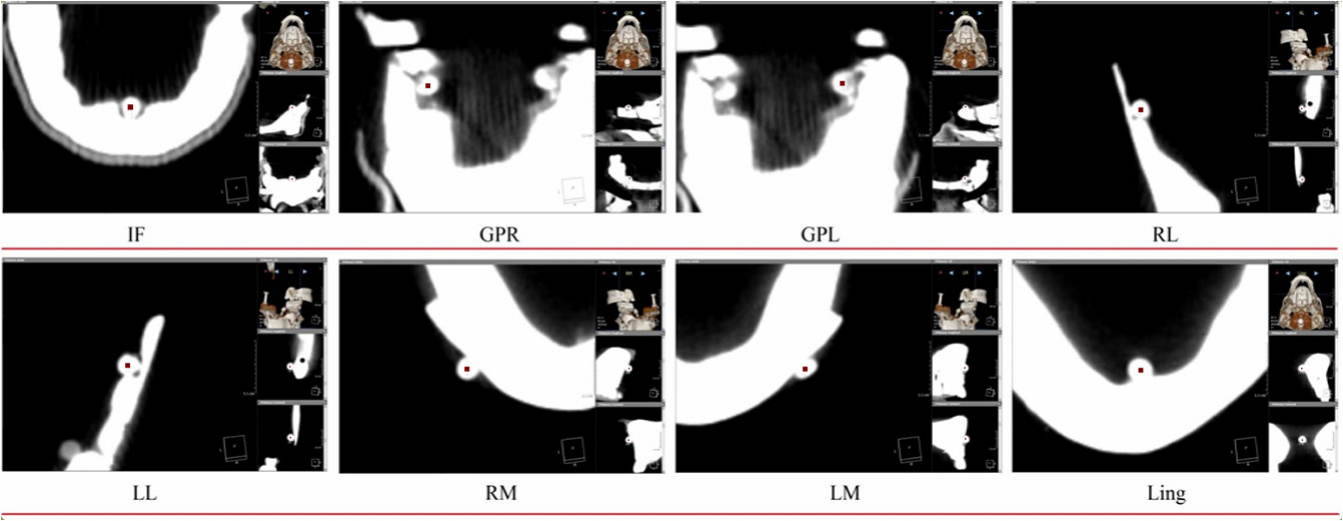
Eight landmarks placed on the DICOM image slice and viewed in 3D: (A) Incisive foramen (IF). (B) Greater palatine right foramen (GPR). (C) Greater palatine left foramen (GPL). (D) Right Lingual foramen (RL). (E) Left Lingual foramen (LL). (F) Right mental foramen (RM). (G) Left Mental foramen (LM). (H) Lingual tubercle (LING).

In total 8 landmarks, 3 in the maxilla and 5 in the mandible were placed. The positions of these landmarks were designed to assess the three dimensional orientation and movements of maxilla and mandible. Each landmark was placed at the centre of the spherical plaster ball placed on the skull prior to scanning as described above. To facilitate onscreen landmark placement, the centre of each sphere was identified by simultaneously viewing the sphere on the DICOM slices in the three dimensions (sagittal, axial and coronal), Fig 2.

Orthogonal measurements of each landmark on the pre-operative slice images to the common reference planes were recorded and the x, y and z coordinates of the 8 landmarks were exported to Microsoft EXCEL for further analyses. The same procedure was repeated for the post-operative slice images for the same case.

### Inter- and intra-examiner’s landmarking error

The inter- and intra-operator reliability of landmark digitisation was assessed by the same operator who digitised the points twice two weeks apart. To assess inter-examiner reproducibility a second independent operator repeated the digitisation of the landmarks. Inter-examiner and intra-examiner landmarking errors were evaluated using a one sample *t*-test and Interclass Correlation Coefficient (ICC). Inter- and intra-examiner landmarking distances errors were calculated using the formula below(14).

$$\text{D}=\sqrt{{\left(\Delta x\right)}^{2}+{\left(\Delta y\right)}^{2}+{\left(\Delta z\right)}^{2}}$$

Where D is the Euclidean distance and x, y and z are the linear measurements in the three respective directions.

The measurements were tested for its reproducibility on a real patients. Four pre- and post-operative DICOM images of patients underwent orthognathic surgery were randomly selected from the database at the dental hospital and school. Ethical approval obtained from the West of Scotland Research Ethics Service on 24th of May 2012 (Rec Reference 12/WS/0133). Each case was landmarked twice by the same observer at two weeks interval. The measurements were compared using paired sample t-test.

### Statistical analysis

The absolute distance between the pre- and post-operative positions of each landmark in x, y and z directions from the DICOM data were compared to the measurements obtained from the simulated orthognathic surgeries using a one sample *t*-test, Interclass Correlation Coefficient (ICC) test and Bland-Altman plot (SPSSVersion22, IBM).

## Results

### Inter- and intra-examiner’s landmarking error

The magnitude of the intra-examiner and inter-examiner landmarking errors are shown in Table 1. The mean landmarking distance errors were 0.35±0.17mm and 0.30±0.15mm for inter- and intra-examiner tests respectively.

**Table 1:** The intra and inter examiner landmarking errors.

Inter-examiner			Intra-examiner		
Landmark	Mean distance (mm)	SD	Landmark	Mean distance (mm)	SD
IF	0.22	0.15	IF	0.21	0.07
GPR	0.29	0.15	GPR	0.13	0.07
GPL	0.38	0.12	GPL	0.25	0.10
LL	0.36	0.19	LL	0.60	0.49
RL	0.39	0.22	RL	0.28	0.08
RM	0.37	0.13	RM	0.25	0.15
LM	0.38	0.30	LM	0.40	0.17
Ling	0.42	0.17	Ling	0.25	0.07

There was a highly significant correlation between the repeated readings for intra- and inter-examiner error test in all three dimensions, Table 2.

**Table 2:** Interclass correlation for the inter and intra-examiner repeated readings.

	Co-ordinate	Mean (mm)	S.D. (mm)	Error Mean (mm)	r-value p-value
Inter-examiner	x	0.02	0.2	0.03	0.63 0.003
	y	0.01	0.17	0.03	0.90 0.001
	z	-0.09	0.7	0.12	0.99 0.001
Intra-examiner	x	-0.04	0.76	0.13	0.99 0.001
	y	0.14	0.32	0.06	0.99 0.001
	z	0.14	0.32	0.06	1.00 0.001

A one sample *t*-test for repeated readings showed no significant difference (p>0.05) in all directions (x, y and z), Table 3.

**Table 3:** The inter and intra examiner errors (one sample t-test).

		Intra examiner error			Inter examiner error		
Landmarks	Coordinate	Mean difference	Standard deviation	p-value	Mean difference	Standard deviation	p-value
IF	X	0.08	1.02	0.88	0.12	0.22	0.34
	Y	0.53	0.99	0.37	0.13	0.17	0.23
	Z	-0.13	0.18	0.23	0.16	0.22	0.24
GPR	X	-0.08	1.06	0.89	0.01	0.36	0.95
	Y	0.62	1.05	0.32	-0.19	0.22	0.18
	Z	-0.15	0.55	0.61	0.23	0.16	0.06
GPL	X	-0.15	1.17	0.82	0.10	0.31	0.57
	Y	0.44	1.01	0.45	0.02	0.23	0.86
	Z	-0.23	0.4	0.33	0.16	0.25	0.28
RL	X	0.26	0.44	0.32	0.08	0.19	0.48
	Y	0.34	0.87	0.49	-0.11	0.17	0.27
	Z	-0.21	0.25	0.19	0.24	0.32	0.23
LL	X	0.10	0.48	0.70	0.06	0.28	0.68
	Y	-0.03	0.59	0.91	-0.04	0.3	0.79
	Z	-0.11	0.17	0.28	0.11	0.18	0.32
RM	X	0.00	0.33	1.00	0.01	0.17	0.91
	Y	-0.08	0.73	0.85	-0.08	0.34	0.66
	Z	-0.05	0.42	0.82	-0.01	0.38	0.96
LM	X	0.72	0.86	0.19	0.16	0.32	0.39
	Y	-0.09	0.48	0.72	-0.11	0.28	0.48
	Z	-0.15	0.46	0.55	0.17	0.28	0.31
LING	X	-0.25	0.55	0.43	-0.04	0.32	0.83
	Y	0.06	0.92	0.91	0.07	0.39	0.76
	Z	-0.14	0.31	0.42	-0.09	0.21	0.44

Accuracy of DICOM slice landmarking compared to the gold standard measurements. There was a significant correlation between the two measurements in both the y and z directions (r = 0.999, p = 0.0001), (r = 0,998, p = 0.0001) respectively, whereas in the x direction there was no significant correlation (r = 0.000, p = 0.500), Table 4.

**Table 4:** The differences between the two methods of measurements (Inter class correlation).

Coordinate	Mean(mm)	S.D (mm)	Error Mean(mm)	Interclass correlation	
				r-value	p-value
X	0.34	0.21	0.03	0.000	0.500
Y	0.08	0.27	0.03	0.998	0.001
Z	0.02	0.22	0.03	0.999	0.000

A one sample *t*-test showed that there was no statistically significant difference for the y and z directions, Table 5.

**Table 5:** The differences between the two methods of measurements (one sample t-test).

Landmark	Coordinate	Mean (mm)	S.D.(mm)	p-value
IF	x	0.35	0.21	.019
	y	-0.01	0.26	0.99
	z	0.06	0.27	0.44
GPR	x	0.33	0.20	.005
	y	-0.15	0.32	0.09
	z	-0.07	0.24	0.27
GPL	x	0.32	0.25	.015
	y	-0.15	0.27	0.05
	z	0.05	0.19	0.28
RL	x	0.43	0.20	.544
	y	-0.25	0.29	0.19
	z	-0.09	0.15	0.34
LL	x	0.29	0.20	.127
	y	-0.18	0.12	0.06
	z	-0.18	0.11	0.05
RM	x	0.32	0.17	.121
	y	0.04	0.12	0.59
	z	-0.08	0.13	0.37
LM	x	0.35	0.16	.164
	y	0.05	0.21	0.70
	z	-0.07	0.25	0.64
LING	x	0.36	0.19	.224
	y	0.11	0.13	0.20
	z	-0.11	0.22	0.40

**Fig 3:**
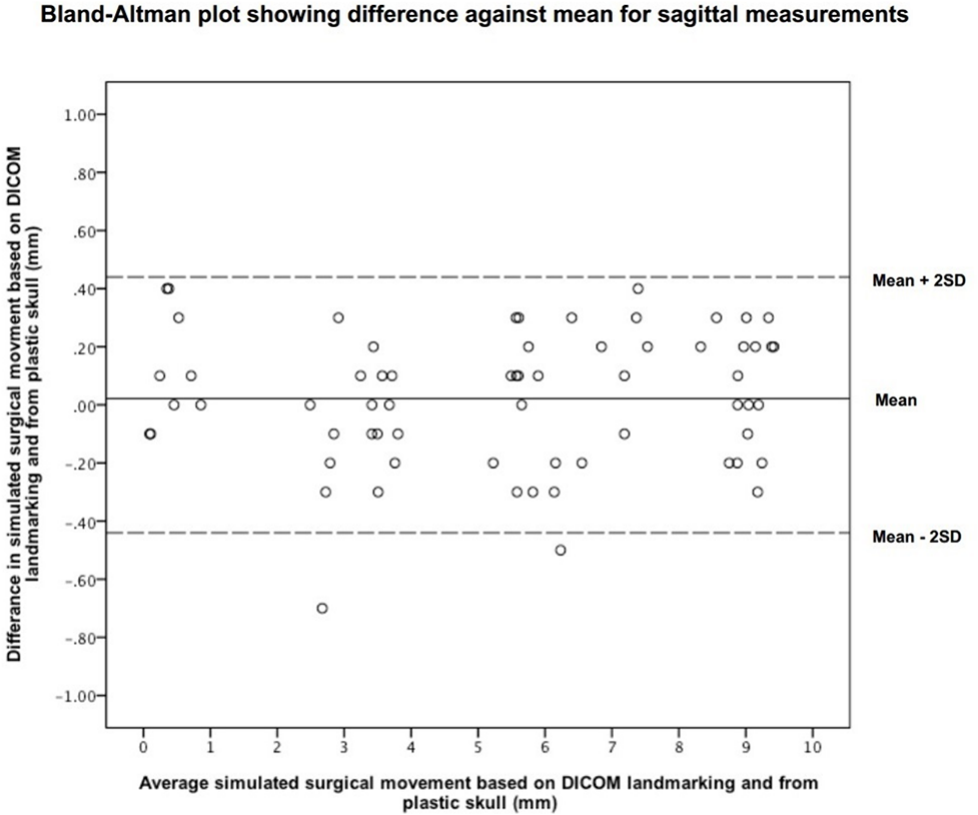
Bland-Altman plot showing the difference against mean in z (sagittal) direction.

**Fig 4:**
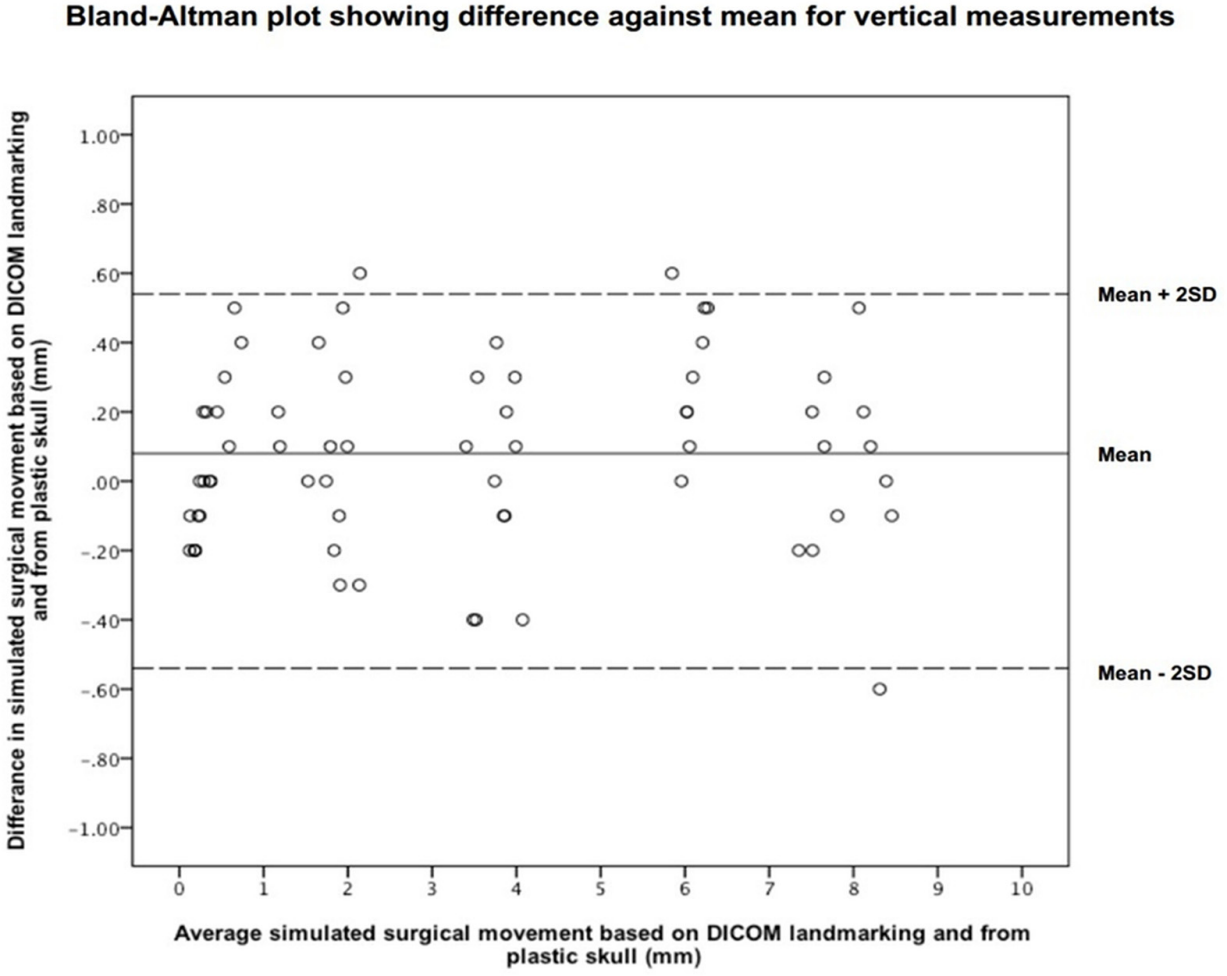
Bland-Altman plot showing the difference against mean in y (vertical) direction.

The x direction, however, showed a significant difference. The mean difference between the two absolute measurements were 0.34±0.20mm, 0.22±0.16mm, 0.18±0.13mm in the y, z and x directions respectively. Fig 3 and Fig 4 show Bland-Altman plots for the sagittal and vertical data.

The results in Table 6 shows a high reproducibility of the measurements on clinical cases. The same landmarks were digitised on the superimposed pre- and postoperative DICOM images. Paired sample t-test showed a low significance values (P = 0.3) in x,y,z dimensions.

**Table 6:** Results of the pilot study of a repeated measurements on clinical cases (Paired sample t-test).

Co-ordinate	Mean(mm)	S.D.(mm)	Error Mean (mm)	p-value	95%CI (lower upper)
X	-0.27	0.77	0.25	0.31	(-0.87 0.32)
y	-0.18	0.62	0.20	0.39	(-0.66 0.29)
z	0.31	0.91	0.30	0.33	(-0.38 1.01)

## Discussion

The primary objective of this project was to introduce and validate a new method of radiographic measurements to assess the maxillo-mandibular changes following orthognathic surgery. Each of the currently available methods has its own deficiency which impacts negatively on the validity of the measurements. The proposed method attempts to overcome the problems associated with the current approaches of 3D surface model segmentation using DICOM. It promises a reliable and a reproducible 3D surface landmarking to measure surgical changes.

Lack of anatomical correspondence is a major problem associated with the colour coded error map method that is frequently used for radiographic superimposition [14]. However, the method is commonly used to evaluate facial soft tissue changes following surgery. It overcomes the difficulty of the limited number of landmarks available for soft tissue analysis. The anisotropic deformation of the soft tissues of the face in response to the surgical movement of the underlying jaw bones is difficult to measure. This is not the case when measuring bony movements of the facial skeleton. As a result of surgery, the maxilla moves as a unified unit, rigid body transformation. The addition of rotational movements such as differential impaction or central midline can produce variation in the amount of linear translation of various regions within the structure. However, this affects the whole skeletal structure as a unit since the geometric integrity is preserved.

Placement of landmarks on a CBCT scan slice is an advanced technique that can provide accurate and detailed information about the internal skeletal structures. This reliability and reproducibility of “slice” landmarking was recently validated[15]. However, the validity of this method to assess skeletal changes following orthognathic surgery has not been tested yet.

Three points in 3D space are enough to create and orient a plane. The coordinates of these three points will be changed as a result of the translation of the plane in 3D space. Rotation of the plane around axes which passes through two of the three points would change the coordinates of the third point, whereas the rotation around any point other than these three points would change the coordinates of the three points. This explains how three points could be used to monitor the position of its related anatomical structure in 3D space. If this plane forms part of a larger solid 3D object then these three points could be used to assess the exact translation and rotation movements of that solid object in 3D space.

This concept was adopted in the proposed approach to assess the skeletal displacement of the maxilla and the mandible as a result of orthognathic surgery for correction of dento-facial deformities. Three anatomical landmarks are therefore required to be marked on each hard tissue structure to accurately measure its displacement in 3 planes of space. To overcome the problems associated with surface remodelling, three landmarks within the maxilla and five landmarks within the mandible were identified directly on the DICOM image slices. Landmark positions on the maxilla and mandible were selected for their favourable geometric position, clear anatomical definitions and high reproducibility.

Simulating the surgical movement on a plastic skull allowed the maxilla and mandible to be separated and translated into different positions anteriorly and vertically with minimal lateral or rotational movements. It was not possible to measure rotational and lateral movements directly on the plastic skull using the current experimental setup; therefore the movement in the x direction was considered to be close to zero throughout the calculations. However the small inadvertent lateral or rotational changes in the x direction led to the expression of a significant p-value and low correlation when compared to the physical movement in the x direction. Differences in measurements in the y and z directions were not statistically significant with a mean difference of 0.22±0.16mm and 0.18± 0.13mm respectively. A one sample *t*-test was used to test for difference from the reference measurement set, the 95% confidence interval was narrow ranging from -0.03 to 0.15mm for the y and z directions, with an upper limit of 0.15mm confirming the clinical insignificance. The high reproducibility of the method on clinical cases validates its applicability in clinical research environment.

The proposed method proved reliable in measuring surgical changes of the jaw bones which was demonstrated by the high correlation coefficient between the physical and digital measurements. The method lends itself to uncomplicated landmarking, the inter-examiner and intra-examiner variability were non-significant

## Conclusions

Internal landmarking of DICOM image slices is a reliable, reproducible and informative method for assessment of the 3D skeletal changes following orthognathic surgery.
